# Distribution of poly(A) polymerase I in bacteria: an expanded role for horizontal gene transfer

**DOI:** 10.1099/mic.0.001755

**Published:** 2026-08-26

**Authors:** George H. Jones

**Affiliations:** 1 Department of Biology, Emory University, Atlanta, GA 30322, USA

**Keywords:** bacteria, horizontal gene transfer, PAP I, polyadenylation, poly(A) polymerase, RNA, RNA decay

## Abstract

Poly(A) polymerase I (PAP I), the product of the *pcnB* gene, catalyses the polyadenylation of RNA 3′-ends in *Escherichia coli*. PAP I and *pcnB* were initially thought to be present only in the β, γ-Proteobacteria and a few other bacterial species. In the present study, blast searches using bacterial proteins bearing the PAP I signature sequence as queries have revealed the presence of proteins containing that signature sequence in a wide range of additional bacterial classes and phyla. Phylogenetic studies indicate that the *pcnB* genes in most of these newly identified species were not inherited by horizontal gene transfer (HGT) from β, γ-proteobacterial donors. Nevertheless, the present studies reveal a larger role for HGT in the inheritance of *pcnB* genes than was documented in previous studies. Together with the other studies cited here, the results presented below strongly suggest that the significant metabolic role for the polyadenylation of RNA 3′-ends in the domain Bacteria.

## Introduction

Polyadenylation of RNA 3′-ends plays a key role in RNA metabolism and function in Archaea, Eukaryotes and Bacteria [1–3]. In the Bacteria, polyadenylation facilitates the degradation of RNA molecules by 3′−5′-exoribonucleases such as polynucleotide phosphorylase (PNPase), RNase II and RNase R [4, 5]. 3′-Poly(A) tails are especially important in facilitating the degradation of structured RNAs, as the tails provide a ‘toe-hold’ for exoribonuclease action [6, 7]. Polyadenylation of bacterial RNAs also plays a role in the regulation of bacterial gene expression. For example, plasmid copy number is controlled by the polyadenylation of regulatory RNAs in *Escherichia coli* and other bacteria. In *E. coli*, polyadenylation regulates the copy number of ColE1 type plasmids [8]. In several species of pathogenic γ-Proteobacteria, virulence determinants are encoded by plasmids whose copy number is regulated by polyadenylation, affecting plasmid maintenance, virulence factor expression and thus the virulence of the pathogen [9]. Polyadenylation is also involved in RNA quality control, policing bacterial RNAs to detect and eliminate transcriptional or processing errors [10]. In a recent study, inhibition of polyadenylation led to the overproduction of free fatty acids in *E. coli* [11]. Several reviews describing the essential features of bacterial polyadenylation have been published [1, 3, 12].

The enzyme responsible for the synthesis of homopolymeric poly(A) tails in bacteria is poly(A) polymerase I (PAP I) [13], the product of the *pcnB* gene (pcn=*p*lasmid *c*opy *n*umber) [13, 14]. PAP I and *pcnB* were originally identified in *E. coli* [13]. In a pioneering study, Martin and Keller used putative PAP I amino acid sequences as queries in blast searches of bacterial proteomes [15]. They concluded that PAP I and *pcnB* were restricted to the β, γ-Proteobacteria, *Chlamydia* and *Spirochaetia*. Of particular significance to the study of PAP I distribution, Martin and Keller identified a signature sequence, [LIV]-[LIV]-G-[RK]-[RK]-F-x-[LIV]-h-[HLQ]-[LIV], which is diagnostic of PAP I enzymes [15].

Using the PAP I signature sequence from various β- and γ-Proteobacteria as queries, Jones performed blast searches of a number of bacterial proteomes [16]. Those searches identified putative PAPs in bacterial species from the α- and ε-Proteobacteria and from four other bacterial phyla, Bacillota (Firmicutes), Actinomycetota, Bacteroidota and Aquificota. Of particular note, the proteins identified in this study were 98–100% identical in amino acid sequence to PAP I proteins found in specific β- or γ**-**Proteobacteria. Additional analyses demonstrated with high probability that the putative PAPs in the species identified in the blast searches arose via the transfer of *pcnB* genes from members of the β, γ-Proteobacteria to ancestors of the relevant species, thus documenting an important role for horizontal gene transfer (HGT) in the inheritance of *pcnB* genes [16].

The availability of an increasing number of genome sequences has made it possible to search the proteomes of additional bacterial species for PAP I. In the present study, blast searches using several PAP I sequences as queries identified putative PAPs in 18 bacterial classes and phyla that had not been known to contain PAP I or *pcnB*. Moreover, the studies presented below document a larger role for HGT in the inheritance of *pcnB* genes than had hitherto been envisioned.

## Methods

### Protein sequences and structure modelling

Amino acid sequences of proteins were retrieved from the National Center for Biotechnology Information (NCBI) protein databases (https://www.ncbi.nlm.nih.gov/). blast searches were conducted using the NCBI blast server with the blastp algorithm with default parameters and the ClusteredNr and Non-redundant protein sequences database. Protein sequences were aligned using M-COFFEE with the default protein alignment parameters [17, 18]. Multiple sequence alignments (MSAs) were displayed using BOXSHADE (https://junli.netlify.app/apps/boxshade/). MSAs are available from the author on request.

Structure homology modelling was performed using SWISS MODEL [19]. Templates were selected from the SWISS MODEL Template Library, and structures were aligned with the COMPARE function of SWISS MODEL. Alignments were viewed and manipulated in PyMol 3.1.4 [20].

### Phylogenetic analyses

Maximum likelihood phylogenetic trees were constructed from the MSAs using PHYML 3.0 (http://www.atgc-montpellier.fr/phyml/) with Automatic Model Selection [21, 22]. The sequences were bootstrapped 1,000 times. The boot trees output file from PHYML was entered into CONSENSE in phylip 3.698 [23] to produce an unrooted consensus tree using M1 as the consensus type and 0.7 as the input fraction. Thus, the consensus trees show only those nodes with bootstrap scores of at least 700. Hillis and Bull demonstrated that bootstrap values of ≥70% generally correspond to a 95% probability that the relevant clade is genuine [24]. The trees were rooted with TreeView 1.6.6 using the *Thermotoga maritima* tRNA nucleotidyltransferase (TNT) sequence as the outgroup. The *T. maritima* sequence was trimmed to eliminate the Nrn (nanoRNase) and CBS (cystathionine β-synthase) domains, which are a part of the native protein sequence but have been shown to have no impact on the catalytic activity of the enzyme [25–27].

### Alien index calculations

Alien indices, indicative of the likelihood of horizontal transfer of *pcnB* genes from a potential donor to a recipient species, were calculated as described previously [28, 29]. The alien index was originally defined by Gladyshev *et al*. [30] and the principle has been refined by Rancurel *et al*. [28]. The latter authors define the alien index as:

AI=ln(best recipient Evalue+1E^−200^) – ln(best donor Evalue+1E^−200^)

The Evalues are determined from blast searches using appropriate query sequences and donor and recipient sequences. In the case of the bacterial PAPs, the query sequence used was that of the PAP whose gene was suspected of acquisition via HGT. The donor species would be the ‘alien’ species, the potential source of the horizontally transferred *pcnB* gene. The recipient species would be one related to the query species, into an ancestor of which the ‘alien’ gene was transferred during evolution. In the context of the present study, potential recipients contain a gene (*cca*) for a TNT but no *pcnB* gene or PAP I protein.

Gladyshev *et al*. proposed an AI value of ≥45 as a strong indicator of HGT. Using a different and larger dataset, Rancurel *et al*. proposed three categories for classifying blast results used in the calculation of the AI: (i) very likely HGT (AI >30 and < 70% identity to candidate donor); (ii) possible HGT (AI >0 and < 70% identity to candidate donor) and (iii) likely contamination (AI >0 and ≥ 70% identity to candidate donor). The stipulation that the query sequence should be <70% identical to the candidate donor sequence only applies to HGT from prokaryotes to eukaryotes and should not affect the analyses presented here [28, 31].

### Other methods

The NCBI Taxonomy Browser was the source of the bacterial class and phylum names utilized in this study, and transcriptome searches were performed using the NCBI Gene Expression Omnibus (GEO) database (https://www.ncbi.nlm.nih.gov/geo/). The geNOMAD programme [32] was used to search candidate genomes for potential mobile genetic elements (MGEs). Genomic sequences were screened for contamination using the Galaxy version (Galaxy Version 0.5.5+galaxy2) of the NCBI FCS-GX contamination detection tool [33]. G+C contents were calculated using the Jamie McGowan GC Content Calculator (https://jamiemcgowan.ie/bioinf/gc_content.html).

## Results and discussion

### blast searches of candidate proteomes identify putative PAPs in additional bacterial taxa

In previous studies, blast searches identified putative PAP I proteins in seven bacterial phyla. However, the provenance of the *pcnB* genes in five of those phyla connects directly to the β, γ-Proteobacteria via vertical or horizontal inheritance [15, 16]. It was of interest in the present study to identify proteins bearing the PAP I signature sequence in species that were unlikely to have inherited *pcnB* genes by HGT from the β, γ-Proteobacteria.

To this end, the PAP I sequences of *Chlamydia pneumoniae* and *Leptospira interrogans* were used as blast queries. Neither the studies of Martin and Keller nor Jones indicated that the *pcnB* genes in these species were inherited by HGT from the β, γ-Proteobacteria [15, 16]. Target phyla for the blast searches were selected based on their positions in the rRNA-based bacterial phylogenetic tree (https://imedea.uib-csic.es/mmg/ltp/) [34]. The species chosen were distributed from the crown to the base of that phylogenetic tree but were otherwise selected essentially at random.

The results of the blast searches are presented in Table 1. Putative PAPs from 18 species, not previously known to contain PAP I or *pcnB*, were identified. Where possible, the taxonomic class (names ending in ‘-ia’ and the Cyanophyceae) for each of the species in Table 1 is indicated. For those species without class assignments, the relevant phylum (names ending in ‘-ota’) is indicated. The species are listed in the order, from crown to base, in which they appear in the rRNA-based phylogenetic tree (https://imedea.uib-csic.es/mmg/ltp/).

**Table 1.** Species containing putative PAP I proteins identified in this study. blast searches were conducted as described in Methods. Proteins IDs are provided for each species along with the taxonomic class or phylum into which each is classified and its abbreviation in parentheses. The closest blast hit for each identified species among the β, γ-Proteobacteria is shown in column 2, with the % identity (column 3) to the PAP identified in column 2. The letter ‘R’ indicates that the sequence of the relevant genome is an NCBI reference genome, whereas the letter ‘M’ indicates that the sequence of the relevant genome was obtained from a metagenome sequencing project

Species	Closest β,γ	% ID
Chrysiogenetes bacterium (Chyb) (Chrysiogenia) (M) MCB0221821.1	γ-Proteobacteria bacterium CAG0950874.1	50.2
Desulfobacterales bacterium (Dsub) (Desulfobacteria) (M) MCJ7785235.1	Burkholderiales bacterium (β) NMA26981.1	41.3
Syntrophobacterales bacterium (Synb) (Syntrophobacteria) (M) MGD8503686.1	Burkholderiales bacterium (β) NMA26981.1	40.0
*Bdellovibrio bacteriovorus* (Bba) (Bdellovibrionia) (M) MGE5153722.1	Chromatiaceae bacterium (γ) MFY9973684.1	89.2
*Enhygromyxa salina* (Esa) (Myxococcia) (R) WP_181232956.1	Burkholderiales bacterium (β) NMA26981.1	44.1
Desulfuromonadales bacterium (Dsb) (Desulfuromonadia) (M) MCK4623112.1	γ-Proteobacterium NIQ12090.1	58.8
δ-Proteobacteria bacterium (Tdsb) (Thermodesulfobacteria) (M) NLV23180.1	γ-Proteobacterium NIQ12090.1	55.7
Verrucomicrobia bacterium (Vrb) (Verrucomicrobiota) (M) MBS0654544.1	γ-Proteobacterium HEY8522058.1	38.3
*Lentisphaera profundi* (Lpr) (Lentisphaerota) (R) WP_274150334.1	Methylococcales bacterium] (γ) MCK5727659.1	34.4
*C. pneumoniae* (Cpn) (Chlamydiia) (R) WP_010892214.1	β-Proteobacterium MGE5126644.1	50.2
*Saltatorellus ferox* (Sfe) (Planctomycetia) (R) WP_145198191.1	*Glaciecola* sp. (γ) MFT4647642.1	55.0
*Luteitalea pratensis* (Lupr) (Acidobacteriota) (R) WP_157898651.1	*Usitatibacter* sp. (β) MHS6181920.1	55.8
Nitrospirota bacterium (Nisb) (Nitrospirota) (M) HET7319729.1	β-Proteobacteria bacterium MGE5173663.1	77.5
Anaerolineae bacterium (Anlb) (Chloroflexota) (M) MGE5195863.1	γ-Proteobacteria bacterium HEX2237238.1	38.4
Armatimonadetes bacterium (Armb) (Armatimonadota) (M) MBM3466081.1	Burkholderiales bacterium (β) NMA26981.1	64.6
Synechococcaceae bacterium (Sycb) (Cyanophyceae) (M) NJM12492.1	Lysobacteriaceae bacterium (γ) HHW76371.1	82.9
*Pedobacter* sp. (Ped) (Bacteroidia) (M) MGE4542720.1	γ-Proteobacteria bacterium NIQ12090.1	57.8
Gemmatimonadales bacterium (Gemb) (Gemmatimonadia) (M) MFC1544005.1	Burkholderiales bacterium (β) NMA26981.1	43.0
*L. interrogans* (Lin) (Spirochaetia) (R) WP_061242599.1	Burkholderiales bacterium NMA26981.1 (β)	37.8
*Syntrophorhabdus* sp. PtaB.Bin184 (Synr) (Syntrophorhabdia) (M) OPY04243.1	γ-Proteobacteria bacterium HSN16644.1	44.4
*Streptococcus pneumoniae* NCTC7978 (Bacilli) (R) VTQ30569.1	*E. coli* (γ) (KZJ88923.1)	99.8

**Fig. 1.**
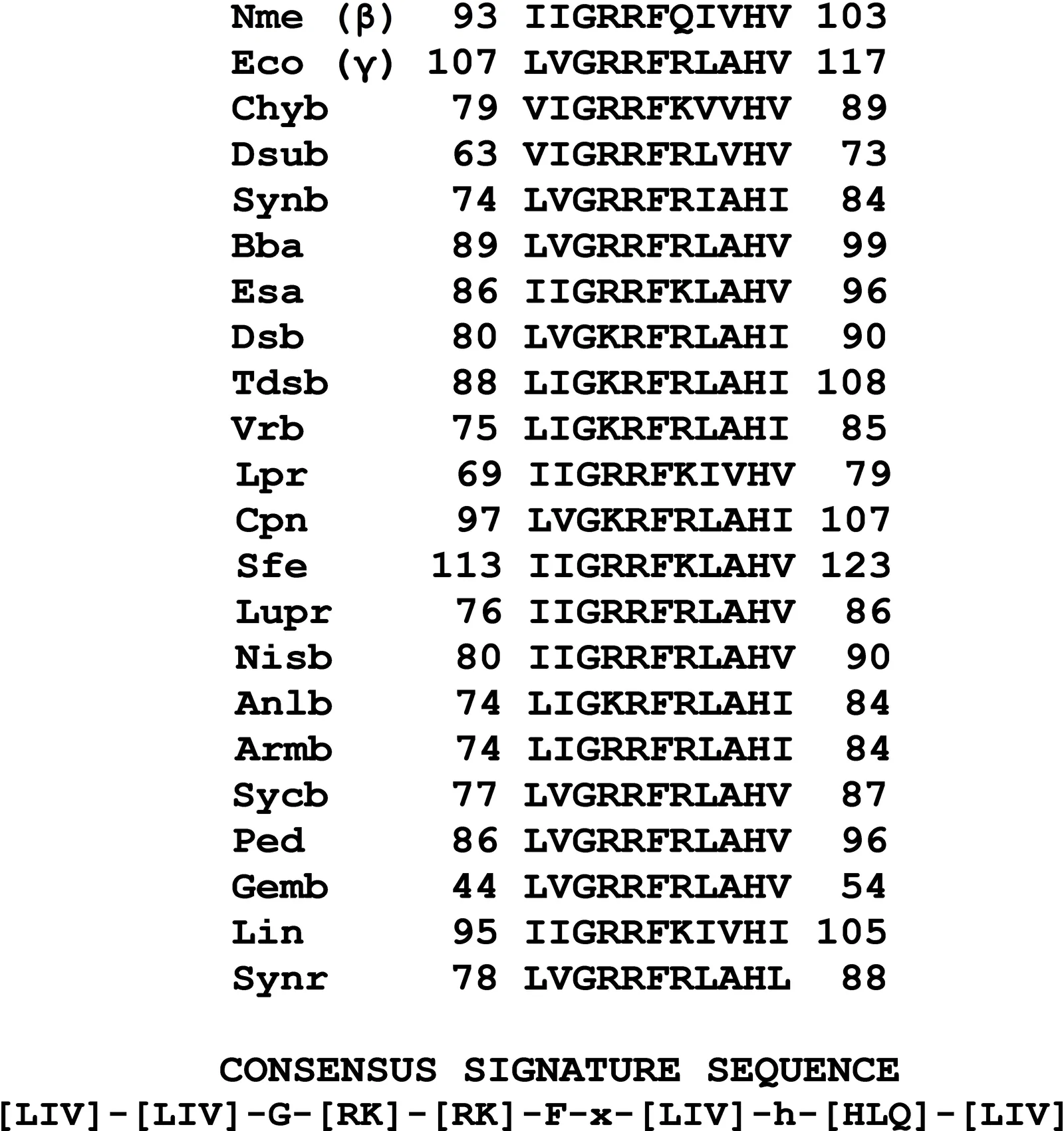
Signature sequences for the putative PAPs identified in this study. Species abbreviations are as indicated in Table 1. The signature sequences of the PAPs from *Neisseria meningitidis* (Nme) and *E. coli* (Eco), representing the β- and γ-Proteobacteria, respectively, are shown for comparison. The consensus signature sequence is shown at the bottom of the figure.

All the proteins identified via blast contained the PAP I signature sequence (Fig. 1), confirming the utility of this sequence in identifying putative products of the *pcnB* gene. Note that, for reference purposes, the putative PAPs from *C. pneumoniae* and *L. interrogans* are included in Table 1 as is the PAP from *Streptococcus pneumoniae*, whose gene was shown previously to have been inherited horizontally from *E. coli* or an ancestor thereof [16].

As an initial attempt to determine the likelihood that any of the putative PAPs listed in Table 1 were inherited via HGT from the β, γ-Proteobacteria, the sequence of each protein in the table was used as a blast query with the proteomes of β, γ-Proteobacteria as targets. The percentage identity of the closest hit from the blast search is shown in the third column of Table 1. In the previous study, the PAP I sequences in the species identified via blast search, e.g.* S. pneumoniae*, were 98–100% identical to those of the potential β- or γ-proteobacterial donor [16]. In contrast, the closest blast hits among the β, γ-Proteobacteria identified in the present study were, with two exceptions, only 35–65% identical to the query sequence (Table 1). The exceptions were *Bdellovibrio bacteriovorus*, whose putative PAP is 89% identical to that of the closest potential donor, a γ-proteobacterium belonging to the family Chromatiaceae, and the Synechococcaceae bacterium, whose putative PAP is 83% identical to that of a γ-proteobacterium. These two species will be discussed in greater detail below.

The strength of the taxonomic inferences which led to the species assignments in Table 1 depend directly on the integrity of the genome sequences in which the putative *pcnB* genes are found. Two approaches were used to ensure the integrity of the relevant genomes. First, the genomes of four of the species listed in Table 1 are NCBI reference genomes (designated by the letter ‘R’ in Table 1), indicating that the genomic sequences are essentially complete and are free of significant contamination [35].

The sequences of the 14 other genomes of the species identified in this study are ‘metagenome-assembled genomes’ (MAGs) and were obtained from metagenome sequencing projects (designated by the letter ‘M’ in Table 1). It has been documented that MAGs are subject to contamination [36, 37], thus it was essential to ensure that the genomic regions bearing the putative *pcnB* genes whose products were identified in the blast searches were free of such contamination. To this end, the genomes of each of the 14 MAGs were examined using the NCBI FCS-GX contamination detection tool [33], on the Galaxy Platform. In each case, the result of the FCS-GX taxonomy report was ‘primary-div,’ indicating that the sequence of the contig containing the putative *pcnB* gene corresponded to the input taxon, viz. the species listed as the source of the *pcnB* gene in Table 1.

It is noteworthy that FCS-GX detected significant sequence contamination in the relevant contigs for two of the species initially included in this study, viz. those from species of Oligoflexia and Fibrobacteria. These sequences were, therefore, omitted from further consideration.

### Other relevant features of the putative PAPs

**Fig. 2.**
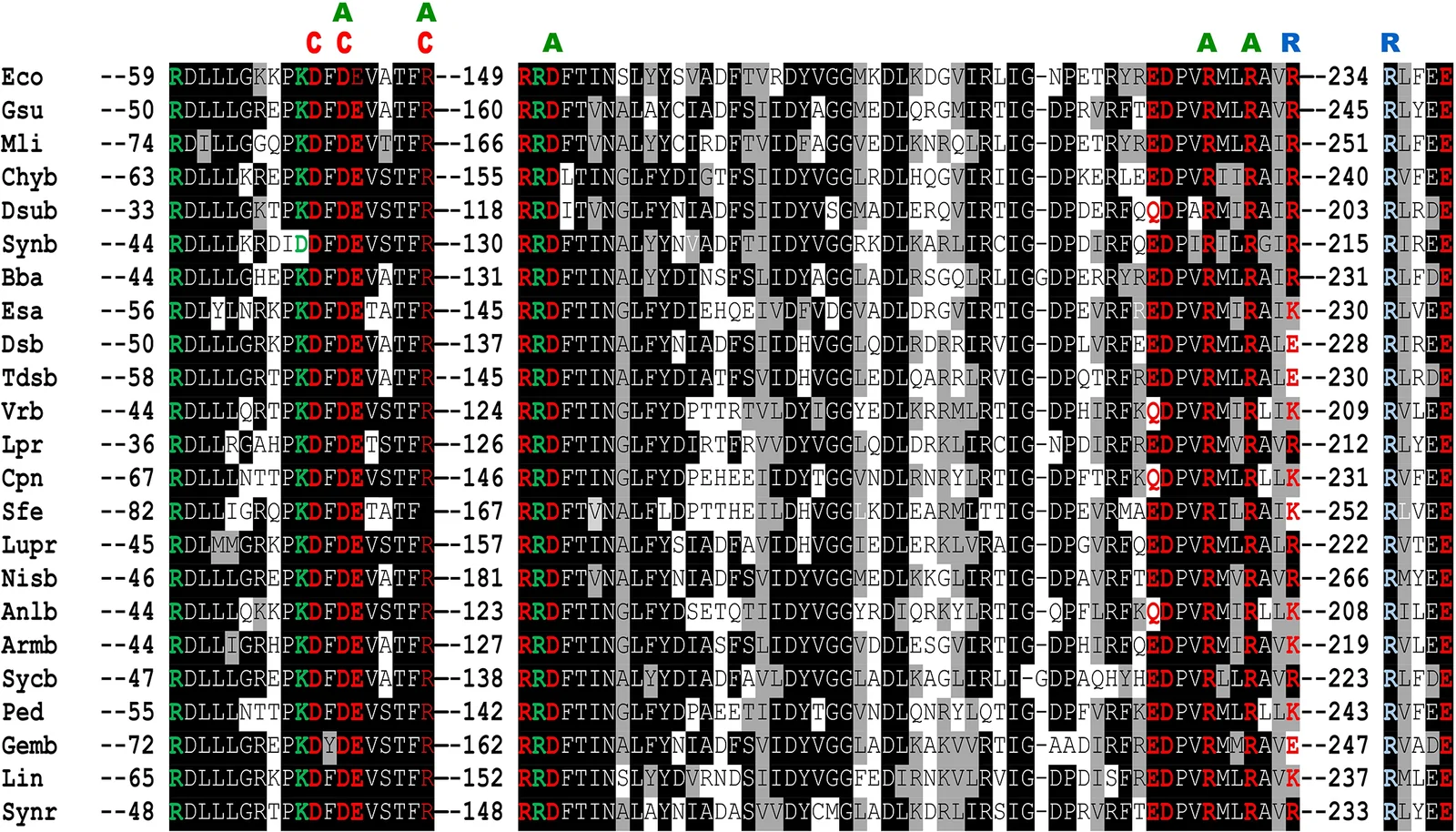
MSA of PAP I sequence regions containing amino acids involved in catalysis and substrate binding. Sequences were aligned with M-COFFEE and displayed using BOXSHADE (https://junli.netlify.app/apps/boxshade/). Identical residues in the various sequences are shaded in black, while similar residues are shaded in grey. In addition to the species listed in Table 1, sequences from *E. coli* (Eco), *G. sulfurreducens* (Gsu) and *M. lipolyticus* (Mli) are shown. Numbers at the left of each block of sequences indicate the position of the indicated N-terminal residue in the PAP I sequence. The three catalytic carboxylates are indicated by the letter ‘C,’ residues involved in forming the catalytic pocket are shown in bold red, those involved in ATP binding are shown in bold green and are designated by the letter ‘A’ and ‘residues involved in RNA binding are indicated in bold blue and are designated by the letter ‘R’. Residues involved in more than one function are indicated by the relevant letter above that residue. The essential residues were identified in the crystallographic analysis of *E. coli* PAP I by Toh *et al*. [38].

As mentioned above, all the proteins identified in Table 1 contained the PAP I signature sequence. Several other features of the proteins and of the species in which they were identified deserve mention. First, all the proteins contain, in addition to the signature sequence, residues that have been implicated in the catalytic activity of PAP I. Toh *et al*. determined the crystal structure of *E. coli* PAP I and identified amino acids involved in catalysis and substrate binding [38]. Fig. 2 shows an MSA highlighting the conserved residues involved in functions identified by Toh *et al*. [38]. In addition to the proteins listed in Table 1, the figure contains PAP I sequences from *E. coli*, *Geobacter sulfurreducens* and *Marinobacter lipolyticus* (see further below).

The three catalytic carboxylates of PAP I are indicated by the red letter ‘C’ above the MSA. Residues involved in forming the catalytic pocket are indicated in bold red, those involved in ATP binding are shown in bold green and residues involved in RNA binding are shown in bold blue. Letters in green and blue indicate residues which were shown by Toh *et al*. to be involved in more than one function. For example, D151 (in the numbering system used by Toh *et al*. for the *E. coli* enzyme) engages in both the formation of the catalytic pocket and in ATP binding [38].

Fig. 2 shows that the essential residues identified in *E. coli* PAP I are highly conserved in the putative PAPs from species listed in Table 1. There are, however, some instances in which substitutions appear in particular sequences. For example, in the Desulfobacteria bacterium, Verrucomicrobia bacterium, *C. pneumoniae* and the Anaerolineae bacterium, glutamine is substituted for glutamic acid at position 193 (E193Q, numbered as in *E. coli* PAP I) [38]. In *Enhygromyxa salina*, the Verrucomicrobia bacterium, *C. pneumoniae*, *Saltatorellus ferox*, the Anaerolineae bacterium, the Armatimonadetes bacterium, the *Pedobacter* sp. and *L. interrogans*, lysine is substituted for arginine at position 203 (R203K). It should be noted that the Verrucomicrobia bacterium, *C. pneumoniae* and the Anaerolineae bacterium contain both the E193Q and the R203K substitutions (Fig. 2). In the Desulfobacteria bacterium, the Thermodesulfobacteria bacterium (listed as a δ-Proteobacteria bacterium in Table 1, see further below), and the Gemmatimonadales bacterium, glutamic acid is substituted for arginine in position 203 (R203E).

Pechmann and Frydman, in an examination of the evolution of protein networks, proposed a classification of amino acid substitutions as conservative or non-conservative based not only on polarity and stereochemistry but also on the impact of specific substitutions on protein stability [39]. Based on their classification system, the E193Q and R203K substitutions are both classified as conservative ones. The K69D substitution, observed in the putative PAP I from the Syntrophobacterales bacterium and the R203E substitution observed in the sequences from the Desulfuromonadales bacterium, the Thermodesulfobacteria bacterium and the Gemmatimonadales bacterium are classified as non-conservative in the system of Pechmann and Frydman [39]. It would be interesting to examine the effects of the non-conservative substitutions on the catalytic activity of the proteins in question.

On the whole, there is significant conservation of the residues implicated by Toh *et al*. [38] as involved in the activity of *E. coli* PAP I among the putative PAPs identified in the present study (Fig. 2).

### Phylogenetic analysis of the PAP I sequences identified in this and previous studies

**Fig. 3.**
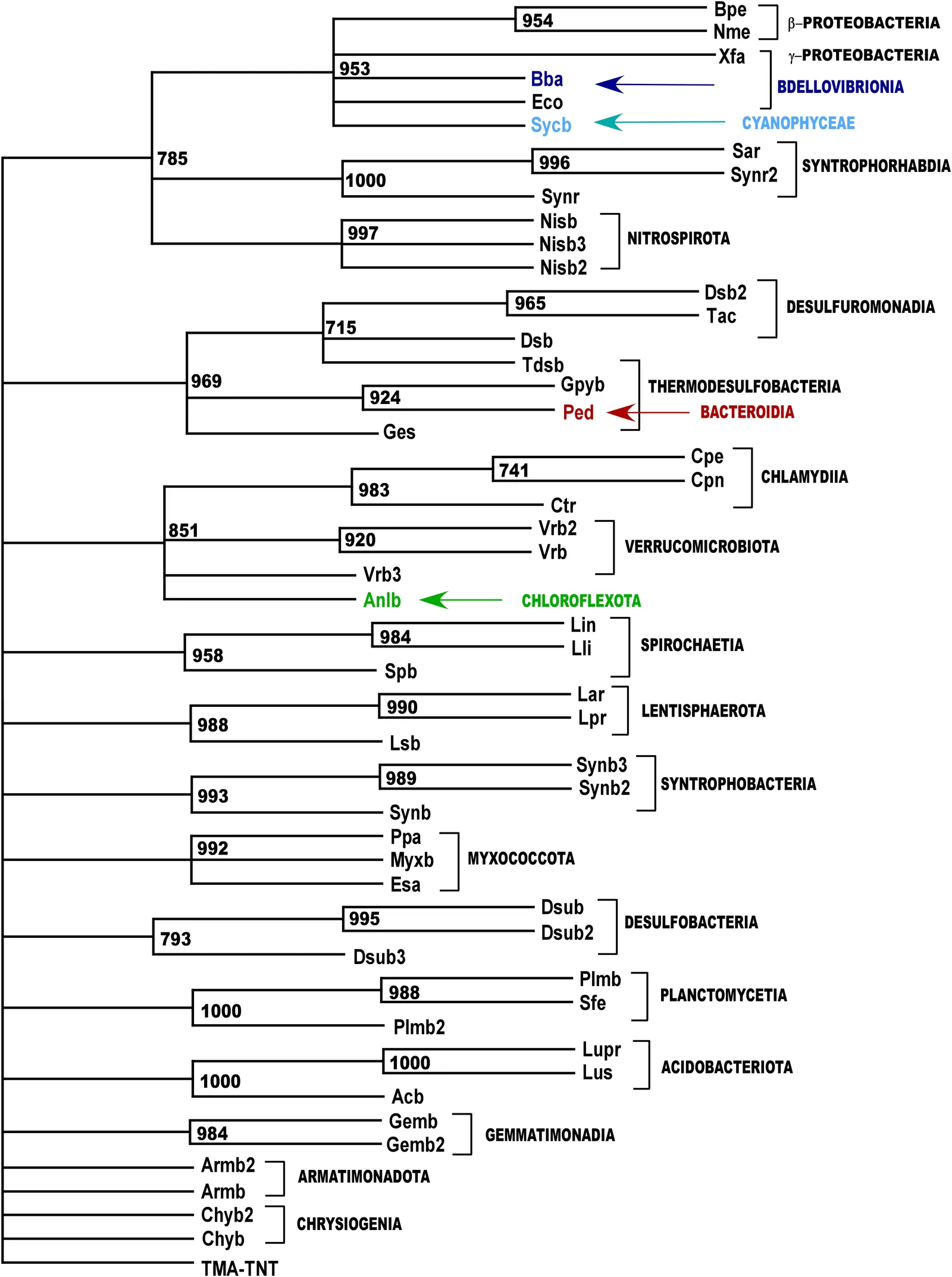
Maximum likelihood phylogenetic tree showing the relationships between PAPs from representatives of the classes and phyla identified in Table 1. Species were selected as described in the text, and the tree includes PAP I sequences from two members of the β-Proteobacteria (*Bordetella pertussis* (Bpe) and *N. meningitidis*) and two members of the γ-Proteobacteria, *E. coli* and *Xylella fastidiosa* (Xfa). Species abbreviations are as indicated in Table 1. PAP I sequences were aligned using M-COFFEE [17, 18] and the phylogenetic tree was constructed using PHYML [21, 22]. The consensus tree was produced from the PHYML boot tree sequences using CONSENSE in phylip 3.698 [23] with M1 as the consensus type and 0.7 as the input fraction.

As an additional step in understanding the evolution of the putative PAP I proteins identified here, phylogenetic analyses were performed. In addition to the sequences listed in Table 1, one or two additional putative PAP I sequences (Table S1, available in the online Supplementary Material) obtained in blast searches for each class or phylum were aligned using M-COFFEE [17, 18], and those alignments were then used as inputs for PHYML 3.0 [21, 22] and phylip [23] as described in Methods. The resulting maximum likelihood phylogenetic tree is shown in Fig. 3.

The tree was rooted using the sequence of the TNT from the bacterium, *T. maritima* from which the NrN and CBS domain sequences were removed. Those domains are not required for the TNT activities of the enzymes in which they are found [25–27]. Several lines of phylogenetic and biochemical evidence suggest that PAP I evolved from an ancestral TNT. First, putative PAP I sequences and sequences from the TNTs of the β- and γ-proteobacteria show significant similarity, especially across the first ca. 25 kilodaltons from the N-terminus of the proteins [40]. Second, as a result of their sequence and structural similarity, bacterial TNTs and PAPs belong to a superfamily of nucleotidyltransferases [41]. Third, biochemical studies have shown that specific mutations in the *cca* gene from *Geobacillus stearothermophilus* converted the TNT from that species into a poly (A) polymerase [42]. Finally, *T. maritima* is situated near the base of the rRNA-based bacterial phylogenetic tree (https://imedea.uib-csic.es/mmg/ltp/) and has been used to root trees involving members of the nucleotidyltransferase superfamily in other studies [16, 29]. It should be noted that the same tree topology was observed when the tree was rooted with the A-adding TNT from *Aquifex aeolicus* [43], a species also situated near the base of the rRNA-based tree (https://imedea.uib-csic.es/mmg/ltp/) (Data not shown.).

The results shown in Fig. 3 argue further against the acquisition of the *pcnB* genes in most of the species shown in Table 1 by HGT from the β, γ-Proteobacteria. Indeed, based on this analysis most of the putative PAPs identified here appear to be more ancient than those of the β, γ-Proteobacteria.

It will be noted that only the single Bdellovibrionia (*B. bacteriovorus*), Anaerolineae, Synechococcaceae and *Pedobacter* sequences from Table 1 were used in the construction of Fig. 3. In preliminary analyses, several additional PAP sequences from the Bdellovibrionia, Chloroflexota, Cyanophyceae and Bacteroidia were used in tree construction (Table S1). However, even in that analysis, the chosen species did not form clades (Fig. S1). The selected sequences were most closely related to PAP sequences from other species rather than to each other (Fig. S1). The likely origin of the *pcnB* genes in the *B. bacteriovorus***,** Anaerolineae, Synechococcaceae and *Pedobacter* species will be discussed in the next section.

One additional point related to species classification should be mentioned here. In previous studies, several PAP and TNT-containing species were assigned to the bacterial class δ-Proteobacteria [16, 29]. Waite *et al*. argued that organisms previously classified as δ-Proteobacteria are not really Proteobacteria at all and proposed reclassification of all the organisms so classified previously [44]. Accordingly, species designated as δ-Proteobacteria in earlier studies are denoted herein as Thermodesulfobacteria (Tables 1 and S1 and Figs 3 and S1).

### The role of horizontal gene transfer in the inheritance of *pcnB* and PAP I

As mentioned, HGT of *pcnB* genes from the β, γ-Proteobacteria has been documented [16]. The hallmark for the identification of HGT is phylogenetic incongruence, that is, the appearance of genes or proteins in clades other than those in which their sister species cluster in phylogenetic trees [45–47]. Fig. 3 shows that the PAP I sequence from *B. bacteriovorus* is most closely related to sequences from the γ-Proteobacteria. The PAP I sequence from the Anaerolineae bacterium is most closely related to PAPs from the Verrucomicrobia, the sequence from the Synechococcaceae bacterium is most closely related to sequences from the γ-Proteobacteria and the *Pedobacter* sp. sequence is most closely related to sequences from the Thermodesulfobacteria. Again, none of the PAP sequences from the Chloroflexota, Cyanophyceae or Bacteroidia formed clades in the phylogenetic analysis shown in Fig. S1, and the sequence of the putative PAP from *B. bacteriovorus* did not appear in the clade containing the sequences from the other Bdellovibrionia in Fig. S1. These results suggest the horizontal transfer of *pcnB* genes to appropriate recipients in these species.

To examine this possibility further, Alien Indices were calculated as described previously [16] and in Methods, with the results shown in Table 2. The potential donor species were a Chromatiaceae (γ) bacterium for *B. bacteriovorus*, a Verrucomicrobia bacterium for the Anaerolineae bacterium, a γ-proteobacterium (Lysobacteriaceae bacterium) for the Synechococcaceae bacterium and a species of Thermodesulfobacteria (Syntrophotalea) for the *Pedobacter* sp. (Table 2 and Fig. 3). The potential recipient in each case was a species related to the Bdellovibrio, Anaerolineae, *Synechococcus* or *Pedobacter* species which contained a TNT but no PAP. AI calculations are presented in Table 2. To verify the utility of this approach, Table 2 also contains positive and negative controls. The positive control is *S. pneumoniae*. As shown previously, sequence comparisons, phylogenetic and G+C content analyses strongly suggested that *S. pneumoniae* inherited its *pcnB* gene by HGT from *E. coli* [16]. The Alien Index calculated for this potential transfer was 378, well above the threshold for likely HGT specified by Gladyshev and Rancurel *et al*. [28, 30]. Thus, the Alien Index calculation provides strong support for the conclusion that HGT from *E. coli* or a close relative gave rise to the *pcnB* gene found in *S. pneumoniae*.

**Table 2.** Alien indices for putative PAPs showing phylogenetic incongruence

Pap-containing species	Potential donor	E value (% identity) (% coverage)	Potential recipient	E value (% identity) (% coverage)	Alien index
*S. pneumoniae* NCTC7978	*E. coli* (γ) PAP (KZJ88923.1)	0 (99.8) (100)	*Streptococcus* sp. 263_SSPC (TNT) (WP_048782664.1)	1e−36 (37.6)(54)	378
*C. pneumoniae*	β-Proteobacterium (PAP) (MGE5126644.1)	5e-82 (50.2) (57)	Parachlamydiaceae bacterium (TNT) (MBA3239383.1)	3e-48 (41.4)(53)	−78
*B. bacteriovorus*	Chromatiaceae bacterium (γ) MFY9973684.1	0 (89.2) (93)	Bdellovibrionales bacterium (TNT) MCB0321368.1	3e-108 (50.7)(75)	213
Anaerolineae bacterium	Verrucomicrobia bacterium (PAP) (MBS0623186.1)	0 (68.4) (95)	Chloroflexota bacterium (TNT) (MGE5218425.1)	1e-41 (39.8)(54)	366
Synechococcaceae cyanobacterium SM1_2_3	Lysobacteriaceae bacterium (γ) (PAP) (HHW76371.1)	0 (82.9) (99)	*Oscillatoria amoena* NRMC-F 0135 (TNT) (MDL5046020.1)	1e-39 (39) (53)	371
*Pedobacter* sp.	Syntrophotalea sp. (PAP) (WP_463312838.1)	0 (83.4) (95)	Flavobacteriales bacterium (TNT) (HIK66686.1)	1e-32 (35.5)(50)	387

The negative control is provided by *C. pneumoniae*, which showed no evidence of HGT in Fig. 3 or S1 and for which evidence for vertical inheritance of the *pcnB* gene had been presented previously [15]. The potential donor in this scenario would be the closest blast hit from the β, γ-Proteobacteria and the potential recipient, a species from the class Chlamydiia containing a TNT but no PAP. This scenario produced an Alien Index of −78, strongly supporting the vertical rather than horizontal transfer of *pcnB* genes in the Chlamydiia.

Table 2 shows the Alien Index calculations for the suspected HGT to *B. bacteriovorus*, to the Anaerolineae bacterium, the Synechococcaceae bacterium and the *Pedobacter* sp. Values of 213, 366, 371 and 387, respectively, were obtained in these calculations. These results, along with the phylogenetic incongruence documented in Fig. 3 and S1, argue strongly for the acquisition of the *pcnB* genes in these species by HGT.

As further support for this conclusion, G+C contents of the *pcnB* genes were calculated and compared with values obtained for the entire genome of the species in question and with the PNPase (*pnp*) genes from that species. There is no available evidence suggesting that *pnp* genes were inherited horizontally [48] in bacteria. The results of this analysis are shown in Table 3, which also includes positive and negative controls. The positive control is again *S. pneumoniae*, whose *pcnB* gene has a G+C content which is virtually identical to that of the potential donor, *E. coli*. The negative control, the *pcnB* gene from *C. pneumoniae*, has a G+C content that is not at all close to that of the potential donor organism.

**Table 3.** G+C content (mol%) of genomes, *pnp* and *pcnB* genes for species posited to have acquired *pcnB* genes by horizontal transfer. The table also includes positive (*S. pneumoniae* NCTC7978) and negative (*C. pneumoniae*) controls for HGT

Species from Table 2	**Genome**	***pnp***	***pcnB***	**Potential donor species**	**Genome**	***pnp***	***pcnB***
*S. pneumoniae* NCTC7978	39.6	44.5	***56.0***	*E. coli*	50.6	54.1	***56.3***
*C. pneumoniae*	40.5	40.5	***37.7***	β-Proteobacterium	66.2	64.0	***67.7***
*B. bacteriovorus*	68.5	65.8	***67.7***	Chromatiaceae bacterium	67.5	64.5	***67.9***
Anaerolineae bacterium	43.0	44.3	***49.3***	Verrucomicrobia bacterium	48.0	50.6	***51.7***
Synechococcaceae bacterium	57.0	59.9	***62.1***	Lysobacteriaceae bacterium	63.5	66.6	***66.6***
*Pedobacter* sp.	53.0	51.3	***59.4***	*Syntrophotalea* sp.	56	54.4	***61.5***

The G+C contents of the *pcnB* genes for all four species identified in Table 1 and Fig. 3, suspected of acquiring those genes by HGT, are generally higher than the corresponding values for their overall genomes or their *pnp* genes and are close, although not identical, to the G+C contents of the *pcnB* genes from the potential donor species (Table 3). The G+C contents analysis is consistent with the hypothesis that the *pcnB* genes in *B. bacteriovorus*, the Anaerolineae bacterium, the Synechococcaceae bacterium and the *Pedobacter* species were acquired by HGT, as the amelioration of horizontally transferred sequences over time to reflect the base composition of the recipient genome is well documented [49].

It should be noted that at least one other species of Bacteroidia inherited its *pcnB* gene via HGT, but in that case, from a member of the β, γ-Proteobacteria [16]. It is also noted that the FCS-GX tool does not flag horizontally transferred sequences as contaminants [33].

### Evidence for mobile genetic elements in the genomes of species suspected of acquiring *pcnB* genes via HGT

In a previous study, the ACLAME programme was used to identify potential MGEs that might be involved in horizontal transfer of *pcnB* genes [50, 51]. The ACLAME website is no longer accessible, thus the geNOMAD computational framework [32] was used to examine the genomes of the four bacterial species indicated above for the presence of plasmid and viral sequences that might serve as vectors for horizontal transfer.

The *B. bacteriovorus* genome sequence contained several plasmid-related sequences upstream and downstream of *pcnB*. For example, a 324 bp sequence related to the plasmid stabilization gene *parE* [52] was situated upstream of *pcnB*. geNOMAD also identified a 1,119 bp sequence related to the type IV secretion system involved in bacterial conjugation [53] in the *B. bacteriovorus* genome. geNOMAD did not identify any viral sequences associated with the *B. bacteriovorus* genome.

In the case of the Synechococcaceae bacterium, a 360 bp plasmid sequence encoding a MobC-like protein, required for mobilization of non-self transmissible plasmids [54], was found 1,176 bp upstream of the *pcnB* start codon. An 1,113 bp viral sequence, encoding various viral functions, e.g. a major capsid protein of the N4-gp56 family [55], was observed, beginning 2,387 bp upstream of the *pcnB* start codon.

In the *Pedobacter* sp. genome, a 40.7 kb plasmid sequence, encoding a number of plasmid functions, including conjugation and antibiotic resistance genes, was observed, beginning 287 kb downstream of the *pcnB* stop codon. The region included sequences similar to those of an integrating conjugative element protein from the PFL_4710 family [56]. The *Pedobacter* sp. genome also contains two provirus sequences, 35.3 and 40.0 kb in length, beginning 123 kb and 196 kb downstream of the *pcnB* stop codon, respectively. While a specific role for the plasmid and provirus sequences in HGT of the *pcnB* gene cannot be verified from these observations, the action of MGEs over genomic distances of several hundred kb has been documented [57, 58].

In the genomes of both the Synechococcaceae bacterium and the *Pedobacter* sp., the viral sequences were similar to those of bacterial viruses from the realm, Duplodnaviria, and the class, Caudoviricetes [59]. These viruses have linear, double-stranded DNA genomes and include viruses such as bacteriophage T4 [59]. geNOMAD did not detect plasmid or viral sequences in the genome of the Anaerolineae bacterium.

Taken together, phylogenetic incongruence, Alien Index and G+C content calculations and the presence of MGEs in the genomes of three of the four species examined suggest that the *pcnB* genes in all those species were inherited by HGT. When considered along with the previous study demonstrating *pcnB* inheritance by HGT from the β, γ-Proteobacteria [16], these observations suggest that HGT played a vital role in the inheritance of PAP I genes in bacteria.

### Evidence for the function of PAP I and expression of *pcnB* in the identified species

The fact that the proteins identified in this and previous studies contain the PAP I signature sequence does not necessarily mean that those proteins function in RNA polyadenylation *in vivo*. There are at least three approaches that can be used to verify the function of the putative PAPs: [1] the proteins can be purified and their catalytic activities verified by biochemical assay; [2] mutations in *pcnB* can be constructed and the impact of those mutations on polyadenylation and other metabolic processes assessed; [3] the transcriptomes of the relevant species can be examined to determine whether the *pcnB* transcript is present *in vivo*. Available literature and the NCBI GEO datasets were scrutinized to determine whether any of the three criteria just listed can be applied to the species listed in Table 1. The results of this analysis are provided in Table 4.

**Table 4.** Evidence for PAP I function and/or *pcnB* expression. Column 1 of the table lists the species examined and their taxonomic classes or phyla. Column 2 indicates whether the PAP I enzymatic activity has been demonstrated for the protein in the species listed in the first column. Column 3 indicates whether the *pcnB* transcript has been detected in the transcriptome of the species listed in column 1. Column 4 indicates whether mutations in *pcnB* have been constructed and, if so, the consequences of such mutations. References for the results indicated in columns 2–4 are provided in parentheses

Species, (class or phylum)	Protein purified and activity assayed *in vitro*	Transcriptional analysis shows expression of *pcnB in vivo*	Mutations constructed in *pcnB*
*E. coli* (γ-Proteobacteria)	*E. coli* PAP I purified and assayed demonstrating poly(A) synthesis [13]	Transcriptional regulation of *pcnB* involves multiple promoters, ppGpp and DksA [77]	*pcnB* null mutation decreased polyadenylation levels by 90% [69]
*Pseudomonas aeruginosa* (γ-Proteobacteria)	–	–	*pcnB* null mutation decreased polyadenylation of 23S rRNA by 80% [64]
*Pseudomonas putida* (γ-Proteobacteria)	PAP I activity purified and assayed demonstrating poly(A) synthesis [61]		
*M. lipolyticus* (γ-Proteobacteria)	PAP I purified and assayed demonstrating poly(A) synthesis [63]	–	–
*G. sulfurreducens* (Desulfuromonadia)	Putative PAP I shown to synthesize poly(A) tails *in vitro* [60]	*pcnB* transcript demonstrated *in vivo* via RT-PCR [60]	–
*B. bacteriovorus* (Bdellovibrionia)	–	RNA-Seq identified a *pcnB* transcript [65]	–
*Myxococcus xanthus* (Myxococcia)	–	Microarray analysis identified a *pcnB* transcript [66]	–
*C. pneumoniae* (Chlamydiia)	–	RNA-Seq identified a *pcnB* transcript [78]	–
*Dehalococcoides mccartyi* (Chloroflexota)	–	RNA-Seq identified a *pcnB* transcript [67]	–
*L. interrogans* (Spirochaetia)	–	RNA-Seq identified a *pcnB* transcript [79]	–

In addition to species identified in this and the earlier studies [15, 16], Table 4 contains information on other species in which PAP I function and *pcnB* expression have been examined. *E. coli* was, of course, the first bacterial species shown to contain the *pcnB* gene and its product and PAP I activity and the expression and regulation of *pcnB* have been verified in that organism (Table 4) [13]. In addition, the Desulfuromonadia species, *G. sulfurreducens*, has been shown to contain a protein that demonstrates PAP activity *in vitro* and a *pcnB* transcript was detected in the *G. sulfurreducens* transcriptome by the Reverse Transcriptase-Polymerase Chain Reaction(RT-PCR) [60]. Perhaps, the earliest characterization of a poly(A) polymerase in bacteria was the study of Payne and Boezi, demonstrating the presence of the enzyme in *Pseudomonas putida* [61]. In a recent study, PAP I was characterized in the γ-proteobacterium [62], *M. lipolyticus* [63].

Evidence was provided in the previous report for the expression and regulation of *pcnB* in *C. pneumoniae* and in *L. interrogans* [16]. Table 4 provides evidence for the function of PAP I and/or the expression of *pcnB* in several other bacterial species. In *Pseudomonas aeruginosa*, a null mutation in *pcnB* decreased the level of polyadenylation of 23S ribosomal RNA [64]. Transcriptional analyses using microarrays or RNA-Seq demonstrated the transcription of *pcnB* in *B. bacteriovorus* [65], *Myxococcus xanthus* [66] and *Dehalococcoides mccartyi* [67]. In each case, the protein encoded by the *pcnB* gene in these species contained the PAP I signature sequence.

Regarding the last point, it is noteworthy that several transcriptome studies examined as a part of the analyses reported here contained transcripts of genes that were annotated as *pcnB*. However, the proteins encoded by those genes did not contain the PAP I signature sequence. It is likely that those genes encoded TNTs and were incorrectly annotated as *pcnB*.

### Distribution and evolution of PAP I and *pcnB*

**Fig. 4.**
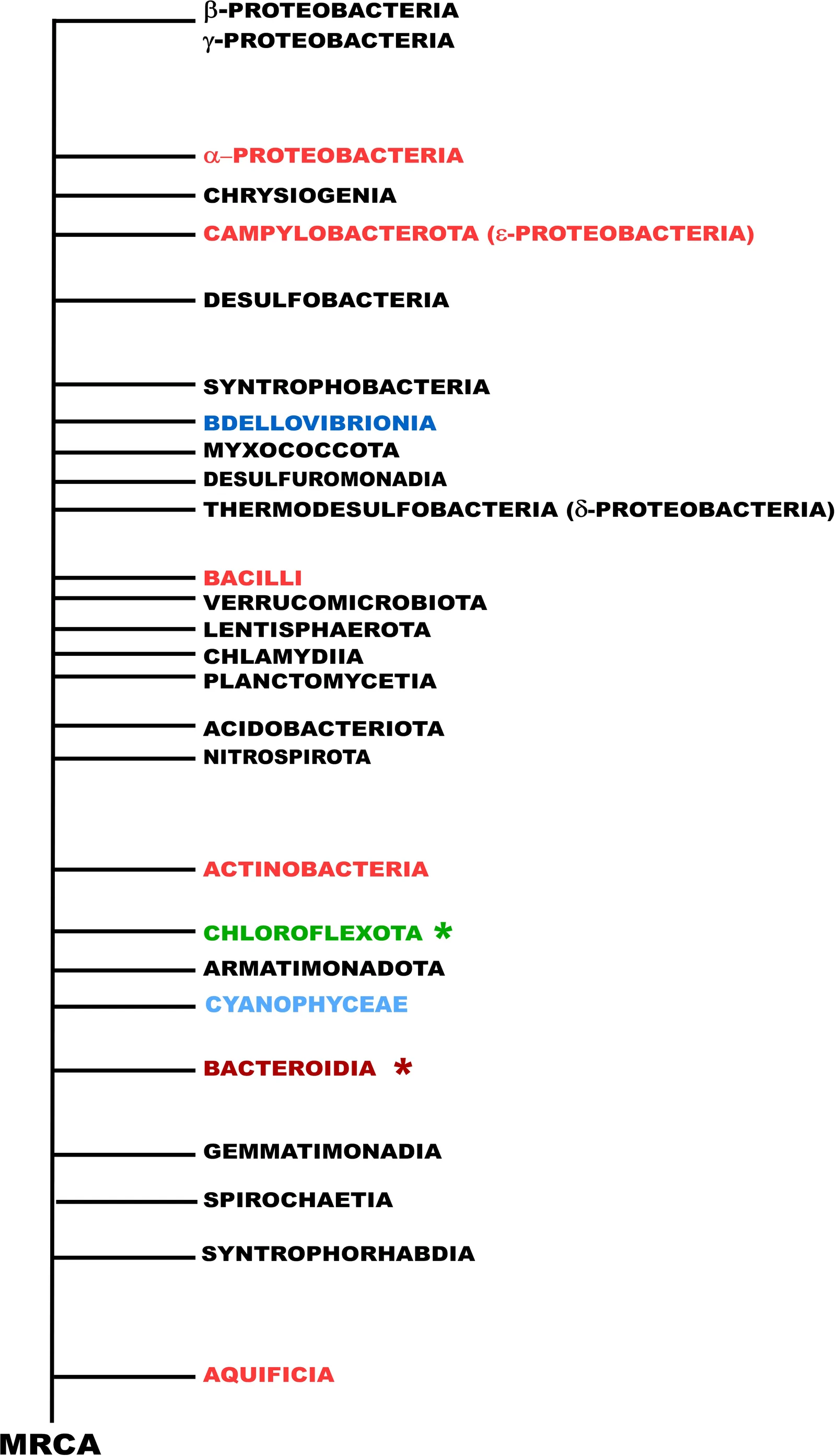
Schematic diagram listing all the classes and phyla shown in this and previous studies to contain proteins bearing the PAP I signature sequence. Classes and phyla are shown in the order, top to bottom, in which they appear, crown to base, in the rRNA-based bacterial phylogenetic tree (https://imedea.uib-csic.es/mmg/ltp/), and the spacing approximates that between classes and phyla in the rRNA tree. The species shown to have inherited *pcnB* via HGT are coloured as in Fig. 3 and Table S1. The two phyla marked with asterisks have acquired *pcnB* genes by HGT from species other than (Chloroflexota) or in addition to (Bacteroidia) the β-, γ-Proteobacteria. MRCA, most recent common ancestor.

The bacterial classes and phyla in which proteins bearing the PAP I signature sequence have been identified are shown in Fig. 4. The classes and phyla are arranged as they appear in the rRNA-based bacterial phylogenetic tree, from crown to base (https://imedea.uib-csic.es/mmg/ltp/). Altogether a total of 27 classes and phyla have been shown to contain putative PAPs, a much larger number than was previously thought [15, 16]. Of this number, evidence has been adduced for the role of HGT in *pcnB* inheritance in nine of these classes and phyla (shown in red, dark red, blue, cyan and green in Fig. 4). These results presumably speak to the importance of RNA polyadenylation in the metabolic economy of bacterial systems.

A scheme for the evolution of PAP I from a TNT ancestor was proposed in an earlier publication [16]. The results presented here are consistent with the essential elements of that scheme but argue for a much greater role for HGT in the inheritance of *pcnB* genes that was considered when the scheme was proposed.

### A modern vestige of a possible ancestor of PAP I

Consideration of possible pathways of PAP I evolution raises an interesting question. Does a vestige of a putative ancestor of PAP I exist in a modern bacterial species? The phylogenetic analyses described above may provide a way to answer that question. The PAP I sequence closest to the root of the phylogenetic tree depicted in Fig. 3 is that from the Chrysiogenetes bacterium. That sequence was used as the query in blast searches of the proteomes of phyla situated near the base of the rRNA-based bacterial phylogenetic tree, viz. Coprothermobacterota, Synergistota, Thermotogota, Dictyoglomota and Caldisericota. These searches identified a protein from a Caldisericia bacterium, protein ID HON84006.1. The protein is 431 amino acids in length and 34% identical, 54% similar in amino acid sequence to the Chrysiogenetes protein over 100% of their lengths. FCS-GX did not report the contig containing the gene for HON84006.1 as contaminated.

Bacteria of the class, Caldisericia, are anaerobic, thermophilic, filamentous, sulfate-reducing bacteria, which because of their novel properties, were assigned to a new bacterial phylum, Caldisericota [68].

Of particular relevance to the present discussion, HON84006.1 contains the following amino acid sequence beginning at residue 58 from the N-terminus of the protein, **P**VGKKFGIV**K**V. It is apparent that, apart from the two residues shown in bold, viz the N-terminal proline residue and the penultimate lysine residue, this sequence conforms identically to the PAP I signature sequence ([16] and Fig. 1). This observation raises the possibility that this sequence may be ancestral to the signature sequences found in modern bacterial PAP I and that HON84006.1 might represent a modern-day vestige of an ancestor of PAP I.

**Fig. 5.**
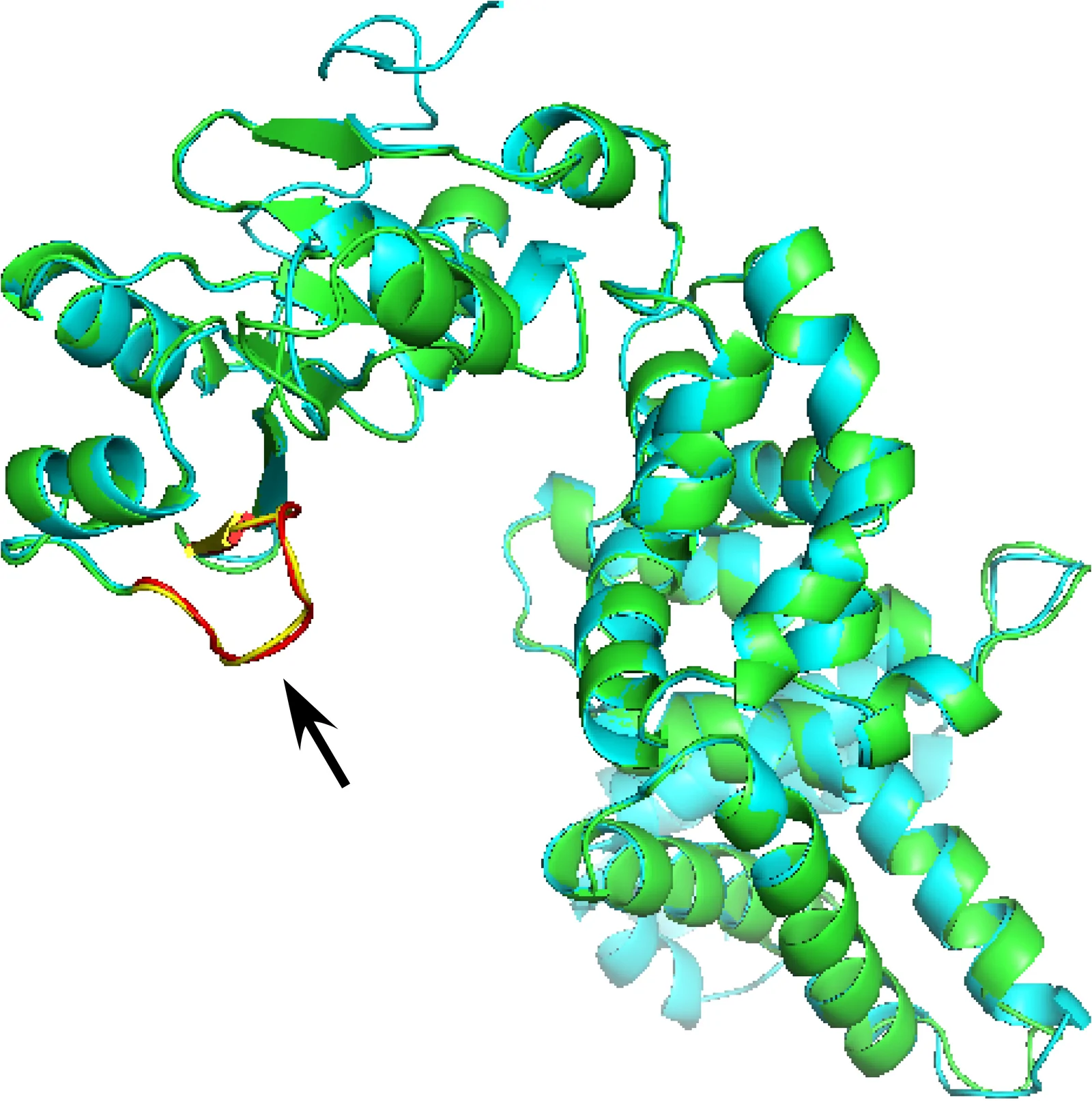
Alignment of the model of Caldisericia bacterium HON84006.1 with *E. coli* PAP I [38]. HON84006.1 was modelled using SWISS MODEL [19], and the structures were aligned with the COMPARE function. *E. coli* PAP I is shown in blue and HON84006.1 in green. The signature sequence in *E. coli* PAP I is depicted in red and the signature-like sequence from HON84006.1 in yellow (arrow).

To explore this possibility further, a structural model of HON84006.1 was constructed using SWISS MODEL [19], with the *E. coli* PAP I structure, PDB ID 3AQL [38], as a template. The two structures were then aligned in SWISS MODEL, and the resulting alignment is shown in Fig. 5. The RMSD for the alignment was 0.390 Å. Inspection of the model shows that the signature sequence in *E. coli* PAP I forms a loop (shown in red in Fig. 5), and that the signature-like sequence from the Caldisericia protein (shown in yellow) aligns precisely with the signature sequence from *E. coli* PAP I.

A blast search using the *T. maritima* TNT as the query revealed that the Caldisericia bacterium contains a protein, ID HOI24540.1, 883 amino acids in length, which is 33% identical and 54% similar in sequence to the *T. maritima* TNT. In addition, HOI24540.1 contains both the Nrn and CBS domains that are characteristic of CCA- and A-adding TNTs found in several bacterial species [25, 26]. Taken together, the foregoing observations suggest the intriguing possibility that the Caldisericia bacterium contains a TNT, HOI24540.1, and a protein, HON84006.1, that is a vestige of the ancestor of modern-day PAP I. It would be of considerable interest to express HON84006.1 and to determine whether the protein possesses enzymatic, particularly polyadenylating, activity.

### Is polyadenylation ubiquitous in bacteria?

As indicated above, blast searches failed to identify putative PAPs in several bacterial phyla, including but not limited to Coprothermobacterota, Synergistota, Thermotoga, Dictyoglomota and Deinococcota. Moreover, although the Aquificia are included in Fig. 4, only one species from that class has thus far been shown to contain a putative PAP and the *pcnB* gene in that species was acquired by HGT from a γ-proteobacterium [16].

Although species in the phyla just listed lack PAP I, RNAs in those species may still possess 3-tails as two other systems for 3′-tail addition are known to exist in bacteria. PNPase has been shown to catalyse the synthesis of heteropolymeric RNA 3′-tails in *pcnB* null mutants of *E. coli* [69]. As PNPase is widely distributed in bacteria [70], it is likely that it serves as the RNA 3′-nucleotidyltransferase in many bacterial species that lack PAP I. Indeed, evidence has been adduced for the function of PNPase in 3-tail synthesis in *Synechocystis* [71], *Streptomyces* [72, 73] and in plant chloroplasts [74]. Moreover, poly(A) tails have been demonstrated in *Bacillus subtilis* and those tails persist in a *pnp* null mutant [75]. As *Bacillus subtilis* does not contain PAP I [76], this species presumably contains a third enzyme system for poly(A) tail addition, apart from PAP I and *PNP*ase.

As more bacterial genome sequences become available, it is likely that additional PAP I-containing species will be identified.

## Supplementary material

10.1099/mic.0.001755 Supplementary Material 1.

## Abbreviations

CBS: cystathionine β-synthase
HGT: horizontal gene transfer
MAG: metagenome-assembled genomes
MSA: multiple sequence alignment
NCBI: National Center for Biotechnology Information
Nrn: nanoRNase
PAP I: poly(A) polymerase I
PNPase: polynucleotide phosphorylase
TNT: tRNA nucleotidyltransferase
