# Supplementary material for: Distribution of poly(A) polymerase I in bacteria: an expanded role for horizontal gene transfer
Source: Microbiology (Reading). 2026 Aug 26;172(8):001755. doi: 10.1099/mic.0.001755 (PMC13514080; doi:10.1099/mic.0.001755)
Supplement: Supplementary file 1 [file mic-172-01755-s001.pdf]

Table S1 – PAP I proteins used in the phylogenetic analysis shown in Figure 3

The table shows the putative PAP sequences used in the construction of the phylogenetic trees shown in Figs. 3 and S1. The additional species from the Bdellovibrionia are shown in blue, those from the Chloroflexota in green, those from the Cyanophyceae in cyan and those from the Bacteroidia in dark red.

SPECIES	PROTEIN ID	% COVER	% ID
**CHRYSIOGENIA**			
Chrysiogenetes bacterium (Chyb)	MCB0221821.1	100	100
Chrysiogenales bacterium (Chyb2)	TFG80055.1	87	37.9
**DESULFOBACTERIA**			
Desulfobacterales bacterium (Dsub)	MCJ7785235.1	100	100
Desulfobacterales bacterium (Dsub2)	MCJ7704581.1	96	72.4
Desulfobacterales bacterium (Dsub3)	NNK95597.1	88	40.5
**SYNTROPHOBACTERIA**			
Syntrophobacterales bacterium(Synb)	MGD8503686.1	100	100
Syntrophobacterales bacterium (Synb2)	MEJ2234547.1	94	75.1
Syntrophobacteria bacterium (Synb3)	MEE9120832.1	99	73.8
**BDELLOVIBRIONIA**			
Bdellovbrio bacteriovorus (Bba)	MGE5153722.1	100	100
Bdellovibrionales bacterium (Bdeb)	MCB0352764.1	57	48.5
Bdellovibrionales bacterium (Bdeb2)	MCB0328730.1	56	47.7
Bdellovibrionota bacterium (Bdeb)	MGA1191720.1	53	48.4
**MYXOCOCCOTA**			
Enhygromyxa salina (Esa)	WP_181232956.1	100	100
Myxococcales bacterium (Myxb)	MCA9682456.1	87	73.3
Plesiocystis pacifica (Ppa)	WP_157595210.1	86	76
**DESULFUROMONADIA**			
Desulformonadales bacterium (Dsb)	MCK4623112.1	100	100
Desulformonadales bacterium (Dsb2)	MDT8419910.1	92	72.8
Trichloromonas acetexigens (Tac)	WP_092057279.1	90	64.3
**THERMODESULFOBACTERIA**			
Deltaproteobacteria bacterium (Tdsb)	NLV23180.1	100	100
Geopsychrobacteraceae bacterium (Gpyb)	HEX9777377.1	91	66.5
Geoalkalibacter subterraneus (Ges)	HDR46091.1	78	63.9
**VERRUCOMICROBIOTA**			
Verrucomicrobia bacterium (Vrb)	MBS0654544.1	100	100
Verrucomicrobia bacterium (Vrb2)	MBS0634438.1	98	68.2
Verrucomicrobia bacterium (Vrb3)	MBS0623186.1	100	53.4
**LENTISPHAEROTA**			

Lentisphaera profundi (Lpr)	WP_274150334.1	100	100
Lentisphaera araneosa (Lar)	WP_007277090.1	96	89
Lentisphaerales bacterium (Lsb)	MCH2206950.1	90	58
**CHLAMYDIIA**			
Chlamydia pneumoniae (Cpn)	WP_010892214.1	100	100
Chlamydia trachomatis (Ctr)	AKR41457.1	97	68.3
Chlamydia percorum (Cpe)	WP_024010726.1	100	76.6
**PLANCTOMYCETIA**			
Saltatorellus ferox (Sfe)	WP_145198191.1	100	100
Planctomycetota bacterium (Plmb)	MFT6110043.1	96	90
Planctomycetota bacterium (Plmb2)	MEM6675190.1	86	76
**ACIDOBACTERIOTA**			
Luteitalea pratensis (Lupr)	WP_157898651.1	100	100
Acidobacteriota bacterium (Acb)	MBA2301315.1	93	62
Luteitalea sp. TBR-22 (Lus)	WP_239491342.1	94	89
**NITROSPIROTA**			
Nitrospirota bacterium (Nisb)	HET7319729.1	100	94.3
Nitrospiraceae bacterium(Nisb2)	MDA8423179.1	91	77.8
Nitrospirota bacterium (Nisb3)	HEX9020871.1	93	76.2
**CHLOROFLEXOTA**			
Anaerolineae bacterium (Anlb)	MGE5195863.1	100	100
Chloroflexi bacterium 13_1_40CM_2_68_14 (Cflb)	OLD45812.1	59	48.6
Dehalococcoidia bacterium (Dhcb)	MCH2516543.1	62	41.8
**ARMATIMONADOTA**			
Armatimonadetes bacterium (Armb)	MBM3466081.1	100	100
Armatimonadetes bacterium(Armb2)	MBI3924907.1	65	68.1
**CYANOPHYCEAE**			
Synechococcaceae cyanobacterium SM1_2_3 (Sycb)	NJM12492.1	100	100
Prochlorococcus sp.(Prch)	GIR74217.1	94	40.9
Kaarinaea lacus (Kla)	WP_455217546.1	90	50.0
**BACTEROIDIA**			
Pedobacter sp. (Ped)	MGE4542720.1	100	100
Sphingobacteriia bacterium (Sphb)	NCA69828.1	97	50.3
Cyclobacteriaceae bacterium (Cyc)	MFT4815867.1	96	49.0
**GEMMATIMONADIA**			
Gemmatimonadota bacterium(Gemb)	MFC1544005.1	90	40.6
Gemmatimonadota bacterium(Gemb2)	MFC1529836.1	82	61.2
**SPIROCHAETIA**			

Leptospira interrogans (Lin)	WP_061242599.1	100	100
Leptospira licerasia (Lli)	WP_010515394.1	97	72.4
Spirochaetes bacterium (Spb)	MBS0618154.1	86	48.9
**SYNTROPHORHABDIA**			
Syntrophorhabdus sp. PtaB.Bin184 (Synr)	OPY04243.1	100	100
Syntrophorhabdus sp. PtaU1.Bin153 (Synr2)	OPY75458.1	93	65.5
Syntrophorhabdus aromaticivorans (Sar)	WP_028895371.1	93	62.5

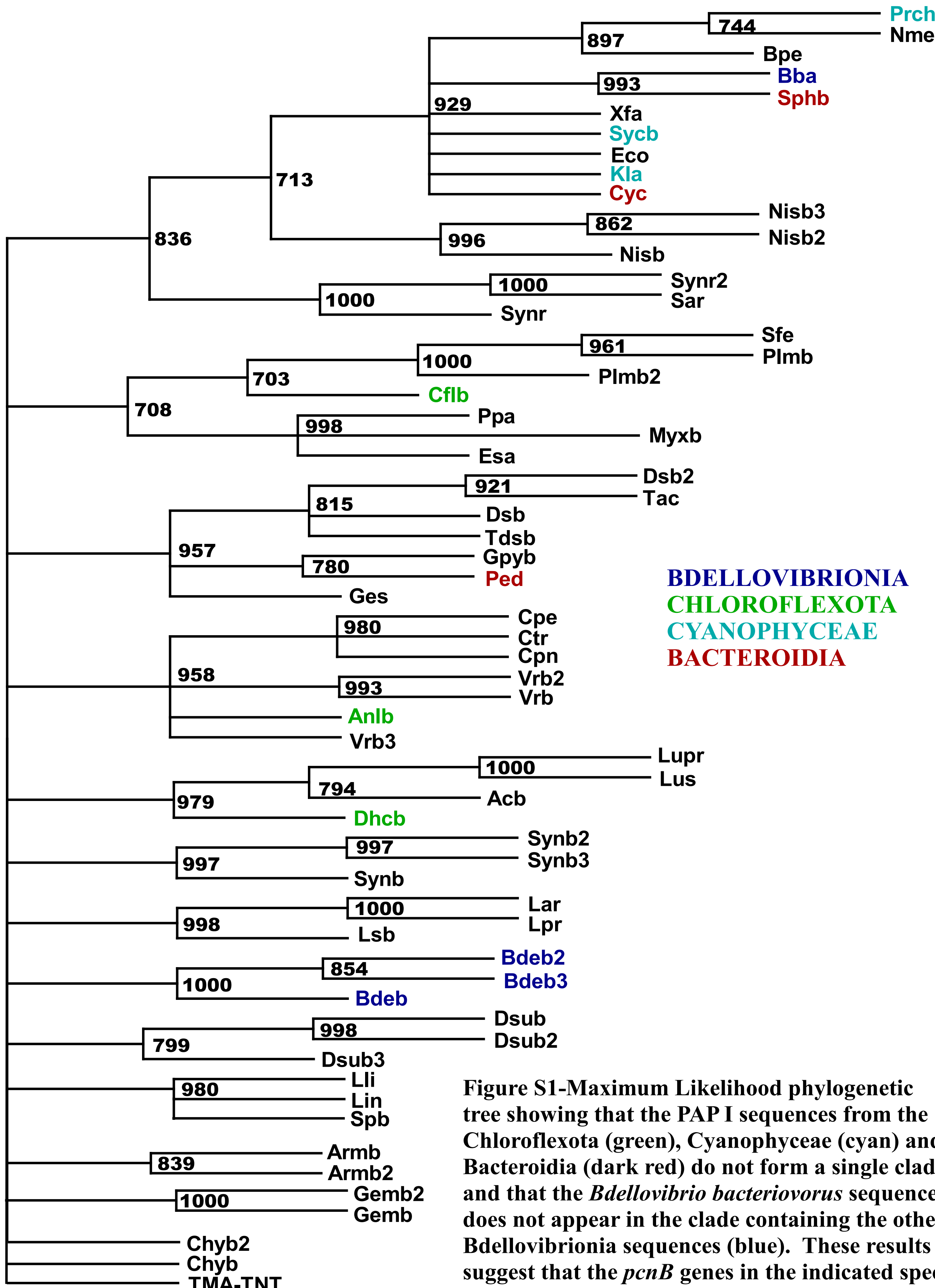

Figure S1-Maximum Likelihood phylogenetic tree showing that the PAP I sequences from the Chloroflexota (green), Cyanophyceae (cyan) and Bacteroidia (dark red) do not form a single clade and that the *Bdellovibrio bacteriovorus* sequence (Bba) does not appear in the clade containing the other Bdellovibrionia sequences (blue). These results strongly suggest that the *pcnB* genes in the indicated species were acquired via horizontal transfer.
